# The Role of Insulin Glargine and Human Insulin in the Regulation of Thyroid Proliferation Through Mitogenic Signaling

**DOI:** 10.3389/fendo.2019.00594

**Published:** 2019-08-28

**Authors:** Xiaoli Sheng, Kannan Yao, Anwen Shao, Sheng Tu, Xinxia Zhang, Ting Chen, Dingguo Yao

**Affiliations:** ^1^Department of Obstetrics, The First Affiliated Hospital, School of Medicine, Zhejiang University, Hangzhou, China; ^2^The Second Central Laboratory, The First Affiliated Hospital of Zhejiang Chinese Medical University, Hangzhou, China; ^3^Department of Neurosurgery, Second Affiliated Hospital, School of Medicine, Zhejiang University, Hangzhou, China; ^4^Department of Infectious Diseases, Collaborative Innovation Center for Diagnosis and Treatment of Infectious Diseases, The First Affiliated Hospital, School of Medicine, Zhejiang University, Hangzhou, China; ^5^Department of Geriatrics, The First Affiliated Hospital, School of Medicine, Zhejiang University, Hangzhou, China; ^6^Department of Ultrasonography, The First Affiliated Hospital of Zhejiang Chinese Medical University, Hangzhou, China; ^7^Department of Endocrinology, The First Affiliated Hospital of Zhejiang Chinese Medical University, Hangzhou, China

**Keywords:** insulin, glargine, thyroid disease, proliferation, insulin receptor

## Abstract

Our aim was to investigate whether human insulin (HI) or insulin glargine treatment could promote the proliferation of thyroid cells and determine the association between type 2 diabetes and thyroid disease. Rats were treated with different doses of HI and insulin glargine. Plasma glucose and the phosphorylation levels of the insulin receptor (IR), insulin-like growth factor 1 receptor (IGF-1R), protein kinase B (Akt), and extracellular signal-regulated kinase 1/2 (ERK1/2) were measured. A total of 105 rats were randomly assigned to three groups as follows: control group, HI group, and glargine group. Both drugs promoted the phosphorylation of IR, Akt, and ERK1/2 in a dose-dependent manner (*p* < 0.05), and the effect of glargine persisted for longer period. Treatment with ultra-therapeutic doses of HI or glargine (*p* < 0.05) increased the expression of Ki-67 in thyroid cells. The results demonstrated that therapeutic doses of glargine have a longer-lasting hypoglycemic control than HI. Based on the results, HI or glargine did not stimulate thyroid cell proliferation at therapeutic doses, but high doses did.

## Introduction

Over the past few decades, it has emerged that diabetes increases the risk of many types of cancer, including thyroid cancer, colon cancer, pancreatic cancer, breast cancer, bladder cancer, and non-Hodgkin lymphoma (1). Among these cancers, thyroid cancer is the most common in diabetic patients. From a 5-years longitudinal study of over 200,000 men and women, it was concluded that diabetes increases the risk of thyroid cancer by 25%, and that the rate of thyroid cancer in women with diabetes is on the rise (2). An endocrine research institute in Copernicus University (Poland) found that patients with type 1 diabetes or type 2 diabetes have a larger thyroid volume than healthy patients, and that the incidence of thyroid diseases (such as thyroid nodules or thyroid tumors) is significantly higher in type 2 diabetes mellitus patients compared to the general population (3).

A study conducted at the American Veterans' Affairs Medical Center suggested that diabetes has a profound impact on the risk of developing thyroid nodules and cancer. Currently, five hypotheses have been proposed to explain the increased risk of thyroid nodules and tumors in diabetic patients. The hypotheses suggest that increased risk of thyroid disease could result from: (1) elevated levels of insulin, insulin resistance, insulin injections, or the use of sulfonylurea drugs; (2) increased BMI; (3) increased thyroid-stimulating hormone levels; (4) chronically elevated blood sugar and triglyceride levels; or (5) vitamin D deficiency (1). Among these hypotheses, previous research has focused on the role of high insulin levels. Diabetic patients have elevated levels of insulin, either due to the injection of exogenous drugs (insulin and its analogs), or as a secondary consequence of resistance to endogenous insulin. Moreover, it should be noted that exogenous drugs play an important role in the late stages of the disease. In the treatment of type 1 and type 2 diabetes, insulin analogs have shown high efficacy and easy application than human insulin (4). However, *in vitro* experiments (5, 6) show that protamine zinc insulin promotes proliferation of breast cancer and bladder cancer cells by increasing phosphorylation of the insulin receptor (IR) and insulin-like growth factor 1 receptor (IGF-1R), also by activating downstream phosphatidylinositol 3-kinase/mitogen-activated protein kinase (PI3K/MAPK) signaling pathways. In 2009, the Diabetologia published four epidemiological studies on diabetic patients and emphasized the potential link between glargine treatment and increased risk of cancer. This concept has caused tremendous controversy in academia regarding the safety of glargine to patients (7–10).

Endogenous human insulin and glargine can bind to IR, and high doses of insulin can also cross-react with IGF-1R. This results in the activation of the PI3K/MAPK signal pathway, which leads to increased metabolism, cell proliferation, and inhibition of apoptosis (11).

Activation of IGF-1R promotes mitosis and reduces apoptosis of tumor cells. These changes are prerequisites to tumor formation. IGF-1R is highly expressed in many cancers, and this is linked to tumor development, invasion, and metastasis. Glargine increases the affinity of endogenous insulin for IGF-1R and enhances its effects on mitosis by 6- to 8-folds (12). Glargine also increases the phosphorylation and activation of known regulators of the insulin-signaling pathway that promote cell proliferation, such as protein kinase B (Akt) and ERK1/2 (13). In this study, we examined the effect of therapeutic doses and supra-pharmacological doses of human insulin (HI) and glargine on the phosphorylation levels of IR, Akt, and ERK1/2 in rats. We also detected the pro-proliferation marker Ki-67 to explore whether HI and glargine promote thyroid cell proliferation.

## Materials and Methods

### Animals

Six to eight-weeks-old pathogen-free Wistar female rats (Slac Laboratory Animal LLC, Shanghai, China) were housed in temperature-controlled environments and fed with standard chow *ad libitum*. All experimental protocols involving animals were conducted in accordance with the guidelines approved by the ethics committee for the use of experimental animals in Zhejiang Chinese Medical University. Blood glucose measurements were performed by collecting blood from tail veins. After blood glucose testing, the thyroid tissues were immediately collected.

### Dosage Groups and Injection of Drugs

Rats were randomly assigned to one of three treatment groups after weighing: control group, HI group, or glargine group (*n* = 35 animals per group). Each treatment group was sub-divided into seven sub-groups, according to the time points; 0, 15, 30, 45, 60, 90, and 120 min (*n* = 5–10 per sub-group). Refer to Emily Jane Gallagher for a description of the grouping method (14). The HI and glargine groups were divided into five groups based in doses: control, 1, 12.5, 50, and 200 U/kg dose (*n* = 5–10). Two weeks before testing, all animals are acclimated to the feeding, injection and fasting protocol for 2 h before injection, but the water was injected in lieu of drug. Injectable drugs were prepared immediately before administration. HI (Novonordisk, Beijing, China) was diluted with saline, while glargine (Sanofi, Beijing, China) was diluted with PBS (pH = 4). Drugs were diluted in 100 μl 0.9% saline or PBS according to the following formula: weight (kg) of rat × experimental dose (1, 12.5, 50, or 200 U/kg) × 10 μl (glargine or human insulin) = mass of drug.

### Protein Extraction

Thyroid tissue from each group was weighed to about 20 μg, and then treated with 200 μl working fluid (adding 1:100 protease inhibitor and phosphatase inhibitors to RIPA buffer inhibitor). The tissue was cut using ophthalmic scissors and homogenized with a tissue homogenizer on ice at 1,500 r/min. After mixing for 30 min, tissue homogenates were centrifuged at 12,000 r/min for 15 min at 4°C. The supernatant was collected.

### Western Blot

The protein samples were separated by 10% sodium dodecyl sulfate polyacrylamide gel electrophoresis (SDS-PAGE), and blotted onto polyvinylidene fluoride membranes (Millipore, USA). All specific antibodies were purchased from Cell Signaling Technology. After blocking in Tris buffered saline and Tween 20 (TBST) containing 5% non-fat dried milk for 2 h, the membranes were incubated with specific antibodies in the following order: IR (1:800, CST 3025), pIGF-1R (1:800, CST, 2969), IGF-1R (1:800, CST, 3027), pErk1/2 (1:1000, CST, 4370), pAkt (1:1000, CST, 4060), Akt (1:1000, CST, 9272), β-actin antibodies (1:1000, BOSTER, BM0627). After 3 washes, the blots were incubated with horseradish-peroxidase-conjugated secondary antibodies at room temperature for 2 h, and visualized with ECL Plus chemiluminescence reagent kit (Beyotime, Shanghai, China) by being exposed to an autoradiographic film (BIO-RAD, ChemiDoc XRS System, USA). Protein expression levels were quantified using Quantity One software.

### Immunoprecipitation

Samples were incubated with magnetic beads under rotation for 2 h at 4°C, after which they were washed with ice-cold RIPA buffer. Antigens were eluted using loading buffer, and then boiled at 96°C for 5 min. The supernatant was collected after centrifugation.

### Immunohistochemistry

Antigen retrieval was performed with high temperature and high pressure using citrate buffer (0.01 M, pH 6.0). Harris hematoxylin was used to stain the nuclei, and ethanol was used for dehydration. The horseradish peroxidase (HRP)-positive cells appeared yellow, with brown granules in the cytoplasm and cell membrane. Image-Pro Plus 6.0 software was used to select a region that acted as a uniform standard to evaluate the positive photos.

### Statistical Analysis

Data were analyzed using one-way ANOVA, as appropriate. All statistics and data analysis were performed using SPSS21.0 software. Data are presented as the mean ± SD, and *P* < 0.05 were considered significant.

## Results

### Glargine Displays a More Prolonged Hypoglycemic Effect Than HI

**Figure 1:**
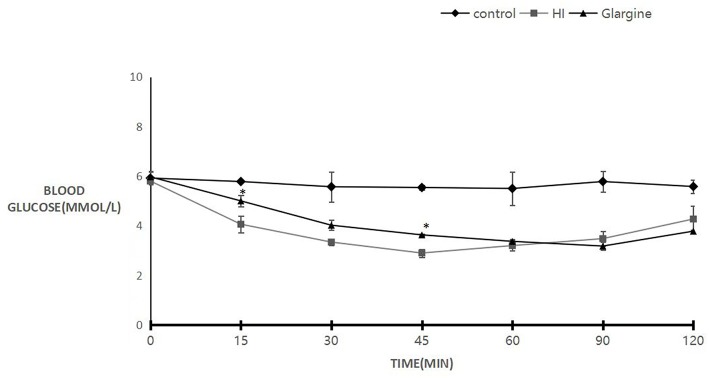
Blood sugar levels of 8- to 10-weeks-old female Wistar rats 120 min after injection with either 0.9% saline (blank group, diamond), 1 U/kg HI (square), or 1 U/kg glargine (triangles) (*n* = 10 rats per group; compared with HI group, ^*^P < 0.05).

Wistar rats were fasted for 2 h before treatment. Blood sugar levels after three treatments were comparable to the levels prior to drug treatment: 5.92 ± 0.25, 5.85, and 5.97 ± 0.23 mmol/L (Figure 1). Subcutaneous injection of 1 U/kg of HI and glargine decreased blood glucose levels rapidly. In HI group, blood glucose levels reached a minimum of 2.90 ± 0.16 mmol/L at 45 min post-injection. Blood glucose in the glargine group reached a minimum level 45 min later than HI group, and was as low as 3.18 ± 0.17 mmol/L at 90 min post-injection. Blood glucose levels in drug treatment groups rebounded after reaching minimum, and the rebound was faster in HI-treated group compared to the glargine group. However, the area under the curve was lower in glargine group compared to the HI group, indicating that glargine produced a more prolonged hypoglycemic effect than HI. At 60 min post-injection, the blood sugar levels in both drug groups were almost similar (3.20 ± 0.21 mmol/L compared with 3.37 ± 0.09 mmol/L), therefore this time-point was used to directly compare the hypoglycemic effects of the two drugs. In comparison, blood sugar levels in the control group (injected with 0.9% normal saline at the same volume) ranged between 5.92 ± 0.25 and 5.50 ± 0.68 mmol/L. Changes in blood sugar in the experimental groups were significantly different at each time point compared with the control group (*p* < 0.05). The difference between HI and glargine group was significantly different at 15 min and 45 min time-points (*p* < 0.01). During the experiment, rats displayed normal levels of activity and no rats died after drug or saline injection.

### Low Doses of Glargine Causes Lower IR Phosphorylation Level and Longer Effect Than HI in Thyroid Tissue

Wistar rats were fasted for 2 h, and then were injected with 1 U/kg of HI or glargine. Previous studies by Hansen et al. (11) and Tennagels et al. (13) indicate that this dose of insulin does not induce phosphorylation of IGF-1R in rat mammary gland or adipose tissue. Therefore, we did not immunoprecipitate IR/IGF-1R, but directly probed for pIR by western blot.

**Figure 2:**
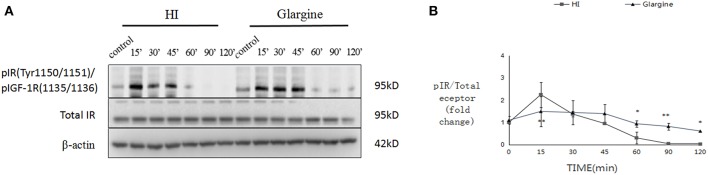
WB images of pIR in rat thyroid cells within the first 120 min after injection of 1 U/kg HI and glargine. Plot of changes of pIR in rat thyroid cells in the first 120 min after injection with 1 U/kg HI (square) or glargine (triangle) as calculated from western blot results (*n* = 5–6; *P < 0.05, **P < 0.01, compared with HI group).

The phosphorylation level of IR in thyroid cells was slightly altered after treatment. In HI group, phosphorylation level peaked at 2.26 ± 0.57-fold change at 15 min, compared to the level of onset, and then decreased rapidly. Phosphorylation levels were close to the limit of detection at 60 min post-injection (Figure 2). Phosphorylation of IR after glargine treatment peaked within 15–45 min post-injection, and then decreased to 40% of the maximum 120 min after injection. The phosphorylation level of IR in thyroid cells was 1.5 times higher than that of glargine, but the peak phosphorylation level was achieved at the same time-point. Similarly, glargine produced a longer-lasting effect on IR phosphorylation compared to HI. HI and glargine had significantly different effects on IR phosphorylation at 15, 60, 90, and 120 min time-points, with glargine group displaying a 2- to 3.1-fold higher IR phosphorylation level after 60 min than HI.

### Akt and ERK1/2 Phosphorylation Are Slightly Higher but Last Longer After Glargine Treatment Compared to HI

**Figure 3:**
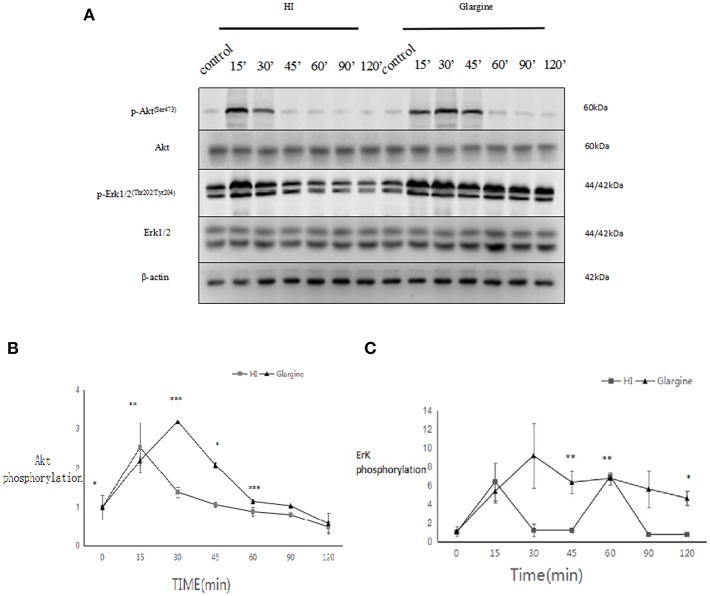
**(A)** pAkt and pERK 1/2 protein levels in thyroid cells of rats injected with 1 U/kg HI or glargine up to 120 min post-injection; **(B)** Plot of changes of pAkt expression in rat thyroid cells within 120 min of injection with 1 U/kg HI (square) or glargine (triangle), as calculated from WB results (*n* = 5–6, *P < 0.05,**P < 0.01, ***P < 0.001 compared to the HI group); **(C)** Plot of changes in pERK1/2 in rat thyroid cells within 120 min of injection with 1 U/kg HI (square) or glargine (triangle), as calculated from WB results (*n* = 5–6).

Rats were fasted for 2 h, after which they were injected with 1 U/kg of HI or glargine. The phosphorylation level of Akt was measured within 120 min. Fifteen min after injection of HI, phosphorylation of Akt reached a peak level, then fell to 19% of the maximum (Figures 3A,B). In glargine group, the peak phosphorylation of Akt reached at 30 min post-injection (15 min later than the HI group), then dropped to 18% of the peak value within 120 min. The results showed that glargine acted on thyroid cells, and that peak pAkt levels were 1.5 times higher after glargine treatment compared to HI treatment. At each time-point, HI and glargine groups were significantly different (^*^p < 0.05, ^**^p < 0.01, ^***^p < 0.001). After 30 min, Akt phosphorylation levels were 1.2–2.3 higher in glargine group than in HI group.

The rats were fasted for 2 h, then injected with 1 U/kg of HI or glargine, and the phosphorylation levels of ERK1/2 were measured. At 15 min post-injection of HI, phosphorylation of ERK1/2 reached a maximum value of 6.39 ± 1.97-fold (which was similar to Akt), then fell and stabilized at lower levels after 30 min, and eventually dropped to 4.63 ± 0.78-folds (Figures 3A,C) after 120 min. The phosphorylation of ERK1/2 peaked at 30 min after glargine injection (15 min later than HI treatment). Thereafter, the levels of pERK1/2 fell slowly, and dropped to 50% of the peak value at 120 min post-injection. These results indicate that glargine acted on thyroid cells, and that the increase in ERK1/2 phosphorylation caused by glargine was 1.43 times higher than that of HI treatment. At 45, 60, and 120 min post-injection, the effects of HI and glargine treatments were significantly different (^*^p < 0.05, ^**^p < 0.01). At 30 min post-injection, ERK1/2 phosphorylation levels were about 7.6–6.2 times higher in glargine group than in HI group.

### High Doses of HI Primarily Affect IR Phosphorylation, Whereas High Doses of Glargine Primarily Affect IGF-1R Phosphorylation

After 2 h of fasting, Wistar rats were injected with 1, 12.5, 50, or 200 U/kg of HI or glargine. At 60 min after injection, the average blood sugar level was 1.8 mmol/L, and there were no obvious hypoglycemic effects, with all rats surviving.

**Figure 4:**
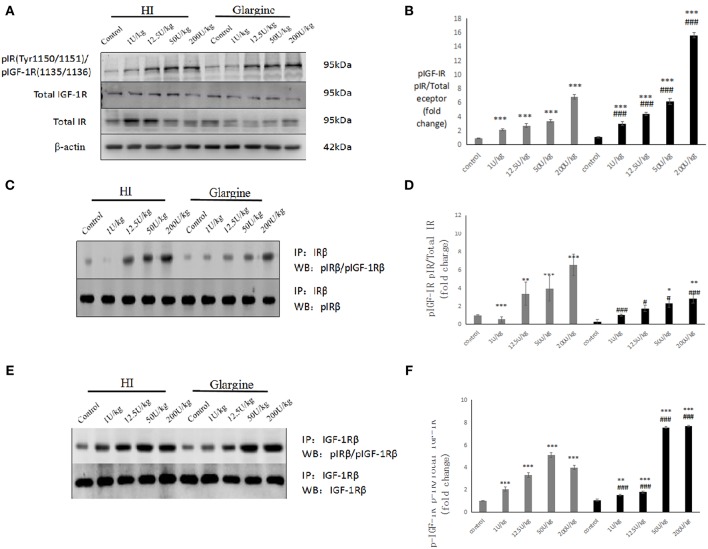
**(A,B)** WB of pIGF-1R/pIR in rat thyroid tissue 60 min after injection of varying doses of HI and glargine. Histogram showing changes in IGF-1Rbeta/IR beta phosphorylation levels in thyroid cells 60 min after injection of 1–200 U/kg of HI (gray) and glargine (black), as calculated from WB results (*n* = 5–7, compared to blank group, *P < 0.05, **P < 0.01, ***P < 0.001; Compared to HI group, ^#^*P* < 0.05, ^###^*P* < 0.001); **(C,D)** Detection of pIR after immunoprecipitation of IR in rat thyroid cells 60 min after injection of different doses of HI and glargine. Histogram showing changes in IR beta phosphorylation in rat thyroid cell 60 min after injection of 1–200 U/kg HI (gray) and glargine (black) calculated from IP and WB results (*n* = 5–8, compared to blank group, *P < 0.05, **P < 0.01, ***P < 0.001; Compared to HI group, ^#^*P* < 0.05, ^###^*P* < 0.001); **(E,F)** WB of pIGF-1R following immunoprecipitation of IGF-1R from rat thyroid tissue 60 min after injection with different doses of HI and glargine. Histogram showing changes in IGF-1R beta phosphorylation in thyroid cells of rats injected with 1–200 U/kg HI (gray) and glargine (black) as calculated from IP and WB results (*n* = 5–7, compared to blank group, *P < 0.05, **P < 0.01, ***P < 0.001; compared to HI group, ^#^*P* < 0.05, ^###^*P* < 0.001).

At each dose, the effects of HI or glargine treatments were significantly different from the control group (^***^p < 0.001) in terms of IR and IGF-1R phosphorylation. In addition, there was a dose-dependent effect of both insulin treatments on the phosphorylation level of IGF-1Rβ/IRβ (Figures 4A,B). HI and glargine treatments were significantly different at the same dosage level (p^***^ < 0.001). At each dose, protein phosphorylation was higher in glargine group than in HI group (1.42-, 1.6-, 1.83-, and 2.28-fold higher for 1, 12.5, 50, and 200 U/kg groups, respectively). Increasing doses of insulin did not have any saturating effect on IGF-1Rβ/IRβ receptors' phosphorylation; even at the highest dose of 200 U/kg, phosphorylation was significantly higher than the other doses, especially in glargine group.

To determine pIGF-1R and pIR cross-reaction, rats were fasted and injected with drugs as described above. Thyroid tissues were collected for each dose. We then used a co-immunoprecipitation approach to calculate the change in IR and IGF-1R phosphorylation.

The IR phosphorylation levels measured at each dose are shown in Figures 4C,D. The phosphorylation levels of IR were significantly different in HI group compared to the control group at each dosage (^**^p < 0.01, ^***^p < 0.001). Rats injected with high doses of glargine (50 and 200 U/kg) had a significantly higher phosphorylation compared to the control group (^*^p < 0.05, ^**^p < 0.01). IR phosphorylation level increased with the drug dose. At the same doses, there were significant differences between HI and glargine groups (^#^*p* < 0.05, ^##^*p* < 0.01, ^###^*p* < 0.001). The phosphorylation level of IR was remarkably (1.71- to 2.3-fold) higher in HI group compared to glargine group at doses above 12.5 U/kg. These findings demonstrate that HI acts on the insulin receptor in thyroid tissue.

According to WB analysis, high doses of glargine caused higher increases in IGF-1Rβ/IRβ phosphorylation than HI. No obvious dose-dependent changes in the phosphorylation level of IR were found in the precipitation analysis. Next, we measured the dose-dependent effects of the drugs on IGF-1R phosphorylation (Figures 4E,F). The IGF-1R phosphorylation levels were significantly different in HI and glargine groups relative to blank control at each dose (^*^P < 0.05, ^**^P < 0.01, ^***^P < 0.001). The phosphorylation levels of IGF-1R increased with insulin treatment dose, with the exception of the highest HI dose, which reduced the phosphorylation level relative to lower doses. At the same dose, HI and glargine produced significantly different effects on IGF-1R phosphorylation (^###^*P* < 0.001). At lower doses (1 and 12.5 U/kg) HI produced a higher phosphorylation than glargine. At higher doses (50 and 200 U/kg) glargine caused higher IGF-1R phosphorylation than HI (~1.48- and 1.92-fold higher) or blank control (7.02- to 7.12-fold higher). We conclude that high doses of glargine increase the phosphorylation of insulin receptors in thyroid tissue.

### Insulin Treatment Has Dose-Dependent Effects on Akt and ERK1/2 Phosphorylation, With Glargine Having Slightly Stronger Effects Relative to HI Group

**Figure 5:**
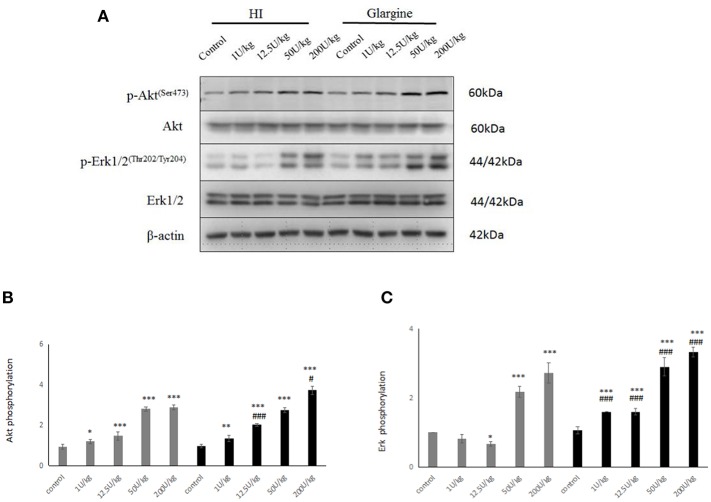
**(A)** WB of pAkt, pERK1/2 in rats 60 min after injection with different doses of glargine and HI; **(B)** Histogram showing changes in Akt phosphorylation in rat thyroid cells 60 min after injection with 1–200 U/kg (gray) and the HI glargine (black), based on WB assays (*n* = 5–6; compared to blank group, *P < 0.05, **P < 0.01, ***P < 0.001; compared to HI group, ^#^*P* < 0.05, ^###^*P* < 0.001); **(C)** Histogram showing ERK1/2 phosphorylation in rat thyroid cells 60 min after injection of 1–200 U/kg HI (gray) and glargine (black), based on WB assays (*n* = 5–8; compared to blank group, *P < 0.05, **P < 0.01, ***P < 0.001; compared to HI group, ^#^*P* < 0.05, ^###^*P* < 0.001.

Having detected changes in the phosphorylation of IR and IGF-1R, we explored changes in phosphorylation of the downstream signaling proteins Akt and ERK1/2. As shown in Figure 5A, the phosphorylation of Akt and ERK1/2 was significantly different at all doses from the control group (^*^p < 0.05, ^**^p < 0.01, ^***^p < 0.001), and the effects followed a dose-dependent pattern. However, it is interesting to note that Akt phosphorylation was similar after treatment with 200 U/kg dose and 50 U/kg dose of HI (2.80 ± 0.10 and 0.10 ± 0.12), indicating the saturation of Akt phosphorylation. At 12.5 and 200 U/kg doses, pAkt levels were higher after glargine treatment compared to HI treatment [1.37 times higher (*p* < 0.05) and 1.29 times higher (^###^*p* < 0.001), respectively]. At the highest dosage (200 U/kg), Akt phosphorylation was 3.8 times higher in glargine than in the control group.

In general, pERK increased with the dose of insulin treatment (Figure 5). ERK1/2 phosphorylation levels in each insulin treatment group was significantly higher than in the blank group (^**^p < 0.05, ^**^p < 0.01, ^***^p < 0.001). At all dosages, glargine caused significantly larger increases in pERK than HI (^###^*p* < 0.001). From lowest to highest dose, glargine treatment increased pERK by 1.95-, 2.39-, 1.34-, and 1.22-fold more than HI.

### Changes in Cell Proliferation and Proliferation-Related Signaling Pathways in Thyroid Cells After HI or Glargine Treatment

**Figure 6:**
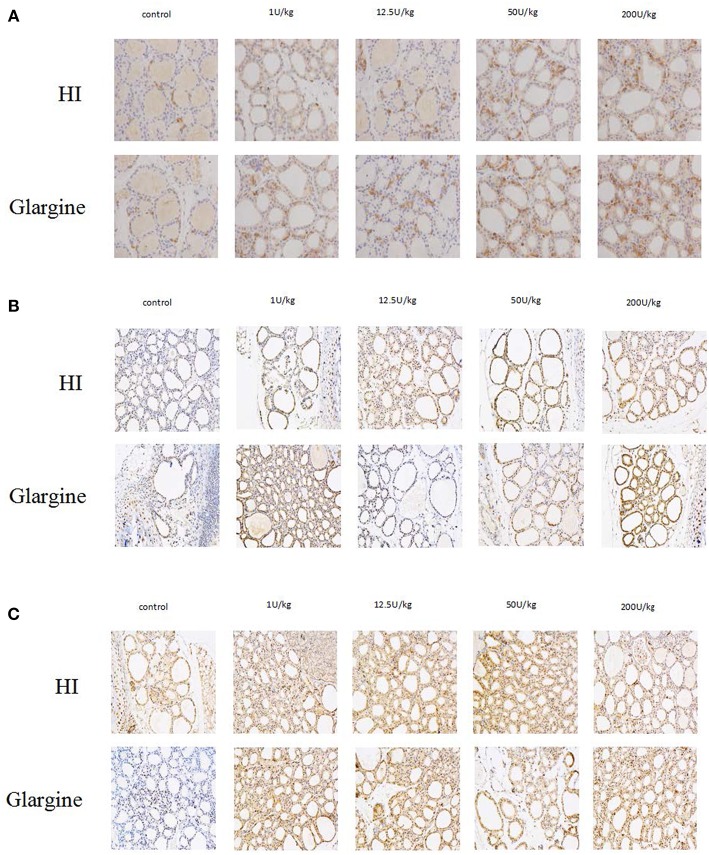
**(A)** Immunolabeling of Ki-67 in thyroid gland cells of rats injected with HI and glargine for 4 weeks; **(B)** Immunolabeling of pAkt in thyroid cells of rat injected with different doses of glargine and HI for 4 weeks; **(C)** Immunolabeling of pERK1/2 in thyroid gland cells of rats injected with varying doses of HI and glargine for 4 weeks.

**Figure 7:**
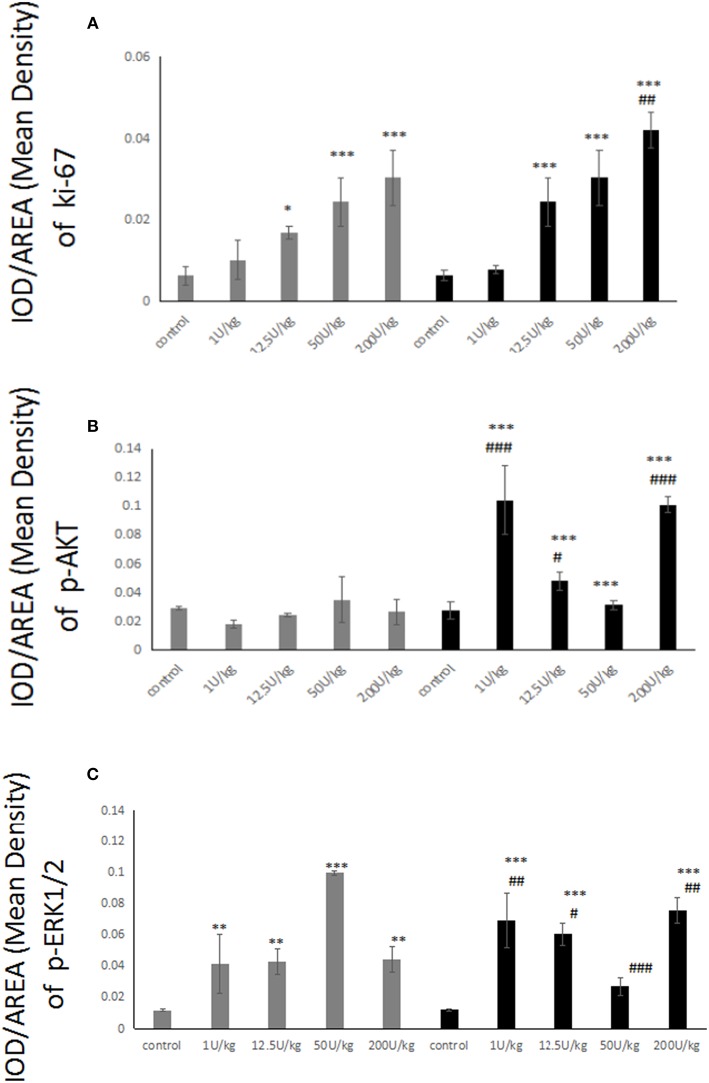
**(A)** Histogram showing Ki-67-positive thyroid cells of rats injected with 1–200 U/kg HI (gray) and glargine (black) for 4 weeks (*n* = 6–10, compared to blank group, *P < 0.05, **P < 0.01, ***P < 0.001; compared to HI group, ^#^*P* < 0.05, ^##^*P* < 0.01, ^###^*P* < 0.001); **(B)** Histogram showing pAkt-positive thyroid cells in rats injected with 1–200 U/kg HI (gray) and glargine (black) for 4 weeks (*n* = 6–10, compared to blank group, *P < 0.05, **P < 0.01, ***P < 0.001; compared to HI group, ^#^*P* < 0.05, ^##^*P* < 0.01, ###*P* < 0.001); **(C)** Quantification of pERK-positive thyroid cells in rats injected with 1–200 U/kg HI (gray) and glargine (black) for 4 (*n* = 6–10, compared to blank group, *P < 0.05, **P < 0.01, ***P < 0.001; compared to HI group, ^#^*P* < 0.05, ^##^*P* < 0.01, ^###^*P* < 0.001).

To explore the effect of high doses of HI and glargine on thyroid cell proliferation, we divided female Wistar rats into HI and glargine treatment groups, and tested the effect of four different doses of the drugs (1, 12.5, 50 and 200 U/kg) 4 weeks after injection (*n* = 10 animals per dose). The highest dose (200 U/kg) had lethal effects; and 40% of rats in this group died. We performed an immunohistochemical examination of Ki-67, Akt, and ERK1/2 in thyroid tissue at 4 weeks after treatment. The results are shown in Figure 6. Round or oval follicles of varying sizes were observed in thyroid cells. Cells were considered positive for Ki-67, Akt, or ERK1/2 if the nucleus appeared brown and cells were yellow and granular. Figure 6A shows that HI and glargine caused a dose-dependent increase in the number of Ki-67 positive cells. The nuclei showing dark staining (positive-cells) were counted and percentage of the total cell population expressing proteins (Ki-67, p-Akt, ERK1/2) was calculated as index (mean density). We found that, in addition to the 1 U/kg dose, intermediate and high doses caused similar increases in Ki-67 as presented in Figure 7A, and the increases were significantly different from the blank group. At the highest doses, HI caused higher increases in Ki-67 expression than glargine.

### Akt and ERK1/2 Phosphorylation Are Slightly Higher but Last Longer After Glargine Treatment Compared to HI

The immunohistochemical labeling of pAkt showed that there were more pAkt-positive thyroid cells in insulin-treated rats compared to rats treated with blank control. The 12.5 U/kg dose of HI group displayed many cells with brown-yellow granules, whereas 1 or 200 U/kg doses of glargine also increased expression, but not in a dose-dependent manner. As shown in Figure 7B, there were no significant differences between any dose of HI and blank control. In contrast, glargine increased pAkt at 1 and 200 U/kg doses to about 3.8 times the level of the blank group. Glargine increased the number of pAkt positive cells more than HI at 1, 12.5, and 200 U/kg doses.

Immunohistochemical labeling of pERK revealed that the insulin treatments produced similar effects; and there were remarkably more positive cells in insulin-treated rats than in the blank group. The 150 U/kg dose of HI group displayed a high number of cells with brown-yellow granules, and there were no differences between various doses of glargine. No dose-dependent trend was observed after HI or glargine administration. As shown in Figure 7C, the differences between the two drug groups and the control group were statistically significant, with higher pERK in the two drug groups, but with no significant dose-dependent trend. Glargine induced higher pERK levels than HI group.

## Discussion

Subcutaneous injection of insulin can maintain glucose utilization at a rate of 1 mg/kg/min for 20 h, both in healthy subjects and in patients with diabetes mellitus. In contrast, glargine absorption is slower and more prolonged compared to human insulin, with no obvious peak (15–17). Several studies have confirmed that (18) when human insulin is absorbed, it primarily binds to the insulin receptor (IR), resulting in IR activation and phosphorylation. Phosphorylation of IR induces random phosphorylation of amino acid residues and activation of downstream signaling targets of IR, including the docking of IRS-1 and IRS-2, activation of signal transduction cascades, which culminates in the regulation of glucose intake and metabolism in skeletal muscle and adipose tissue. Other signaling pathways affected include phosphatidyl inositol kinase (PI3k)/Akt and MAPK. The primary signal transduction mechanism is through activation of PI3K/Akt. At high concentration, insulin can bind to IGF-1R, causing phosphorylation and activation. In general, IGF-1R is activated by IGFs under physiological conditions, and is widely expressed throughout the body. Phosphorylation of IGF-1R results in strong activation of the Ras/Raf/MAPK signal pathways. Gallagher et al. (14) investigated the effects of HI and glargine on transgenic mouse models of breast cancer, and found different levels of phosphorylation of IR, IGF-1R, Akt, and ERK1/2. Tennagel et al. (13) found that HI and glargine caused IR, IGF-1R, and Akt phosphorylation in skeletal muscle, fat, liver, and cardiac muscle in male Wistar rats. However, this study mentioned above did not test the effects on ERK1/2, and changes in signaling pathways compared to mammary gland epithelial cells. Our experimental results show that when HI or glargine acts on thyroid cells, it causes similar changes to those observed in mammary epithelial cells. The biological effect of insulin on target cells depends on its local concentration, the target cell properties, and IR/IGF-1R expression. The similarities observed between thyroid cells and mammary glands may be due to the small number of IR and IGF-1R in these cells, or similar local drug concentrations in these tissues. In addition, the phosphorylation of IR, IGF-1R, Akt, and ERK1/2 in thyroid cells mirrors the curves of hypoglycemic effect of HI and glargine.

*In vitro* experiments showed that glargine can activate IR, and that the downstream signaling pathway is similar to that of human insulin. However, amino acids in the B chain of glargine have been substituted, altering its molecular structure, which accounts for the long-lasting hypoglycemic effect of glargine and increases its affinity for IGF-1R (19, 20). Multiple *in vitro* experiments show that glargine can promote the proliferation of fat cells and breast cells by phosphorylating IGF-1R (21–23). Using immunohistochemistry and qRT-PCR, Liu et al. (24) found that IGF-1 and IGF-1R are up-regulated in thyroid tissues of patients with follicular adenoma, thyroid nodules, papillary thyroid carcinoma, and follicular thyroid cancer relative to healthy controls. Compared to patients with thyroid disease, papillary thyroid carcinoma patients had the highest expression level of IGF 1 and IGF-1R protein and mRNA.

IGF-1 plays an important role in the formation and development of thyroid nodules, including thyroid cancer and thyroid adenoma. As the human insulin analog, insulin B10 aspart (AspB10) is named after its histidine substituted for aspartic acid in the No. 10 B chain. Insulin B10 aspart has higher affinity for IR and IGF-1R than human insulin, and the duration of binding to IR is longer. In addition, insulin B10 aspart has stronger proliferation-promoting effects on mammalian cells than human insulin. In 1992, Drejer (25) tested the effects of AspB10 on female Sprague Dawley rats. Rats were injected with 12.5, 50, or 200 U/kg doses of AspB10, while controls received either saline or 200 U/kg of insulin injections. After 52 weeks, AspB10 caused dose-dependent increases in the incidence of breast cancer, while the control group had no observable increase in breast cancer. After 48 weeks, 44% of rats treated with 200 U/kg of AspB10 had benign breast tumors and 23% had malignant tumors. This study led to the withdrawal of insulin B10 aspart prescription. Although the mechanism by which insulin B10 aspart increases the risk of breast cancer is not clear, many studies (21–23) have proposed that this may be due to the high affinity of insulin B10 aspart for IGF-1R. Thus, researchers speculate that similar insulin analogs that show high affinity for IGF-1R *in vitro* may have pro-proliferative and cancer-causing effects. In this study, when female Wistar rats were treated with 12.5 U/kg glargine for 4 weeks, we observed a significantly higher number of Ki-67 positive thyroid cells compared to control rats. The proportion of Ki-67-positive cells in thyroid cancer tissues was 100%, and the proportion was 30% in benign thyroid lesions. Therefore, our results suggest that treatment with high doses of HI and glagine for 4 weeks causes thyroid cell proliferation. According to the immunoprecipitation and WB analysis, the proliferation-promoting effects of glargine may be associated with activation of IGF-1R, which is consistent with findings from previous *in vitro* experiments (21–24). In clinical practice, a low dose of 1 U/kg is used. Although this dose of HI and glargine induced higher phosphorylation of IR and IGF-1R than the control after 60 min, it did not increase thyroid cell proliferation (as measured by Ki-67-positive cell quantification) after 4 weeks of treatment. Currently, no experiments have been reported on whether extending the time of drug intervention could promote cell proliferation, or whether low doses of HI and glargine could increase the number of Ki-67-positive thyroid cells after 52 weeks of treatment. In our study, we demonstrated that both HI and insulin glargine affect the proliferation of cells in a dose-dependent manner. However, in patients treated with daily doses (0.2–0.4 IU/kg) of insulin glargine, the plasma insulin concentrations remain between 50 and 200 pmol/L or sometimes reach a slightly higher level ranging from 100 to 500 pmol/L (26, 27). The high dose of 200 IU/kg in our study that resulted in high levels of cell proliferation is rather supraphysiological. While therapeutic doses of HI and glargine do not cause thyroid cell proliferation. The published RCT ORIGIN explore effect of glargine insulin on cancer, showed that insulin glargine have a neutral association with overall and cancer-specific outcomes, including cancer-specific mortality. The results also demonstrated that exposure to glucose-lowering therapies, including metformin, and HbA1c level during the study did not alter cancer risk (28).

It is worth noting, however, that high doses of HI increase thyroid cell proliferation. *In vitro* experiments have shown that HI can promote the proliferation of thyroid follicular cells and human endometrial cells, and can increase malignant transformation of human endometrial cells (29, 30). Research into the mechanism behind this has focused on the effect of HI on IR. There are two subtypes of IR (IR-A and IR-B), and the distribution and biological effect of these subtypes are different (31). IR-A expression is increased during thyroid cancer infiltration (32). Pandini et al. (33) found that thyroid cancer tissues with low levels of differentiated cells and high numbers of undifferentiated cells have higher expression levels of IR-A. Therefore, at the start of treatment with insulin and insulin analogs, high number of IR-A are phosphorylated and activated, which may play an important role in the process of cancer development and progression. Pandini et al. (34) postulated that the combination of increased insulin and IR-A activation can increase the expression of the vascular Mrp/PRL gene and cell proliferation protein to promote cell proliferation. A study by Gallagher et al. (14) showed that HI promotes the growth of mammary gland cells and breast tumor proliferation via IR phosphorylation, rather than IGF-1R phosphorylation. In this study, we focused on thyroid cells. Analysis of Ki-67 expression by immunoprecipitation, WB, and immunohistochemistry in thyroid cells produced similar results. HI causes IR phosphorylation in thyroid cells in a dose-dependent manner, which may be due to the increase in thyroid cell proliferation at high doses of HI. There was no significant effect of IGF-1R phosphorylation on thyroid cells after high doses of HI. Therefore, it is not clear whether thyroid cell proliferation is increased through IGF-1R phosphorylation following HI treatment.

As mentioned above, HI and its analogs activate the PI3K/Akt and MAPK signaling pathways. PI3K/Akt signaling increases the number of cells by inhibiting cell apoptosis, which plays a key role in modulating the effects of insulin and IGF-1 on metabolism and mitosis (35). MAPK signaling is associated with increased DNA synthesis and cell proliferation (18). In this study, HI and glargine promoted Akt phosphorylation with increasing doses and time after administration. pAkt levels were slightly higher after HI treatment than glargine. Müller et al. (36) studied the effect of glargine and HI on rat thyroid cell lines FRTL-5 and thyroid follicular cells, and found that the two drugs enhanced the phosphorylation of PI3K/Akt. Our results are in agreement with these findings. There is also evidence that HI treatment increases cell proliferation via Akt activation in bladder cancer cells, breast cancer cells, and rat fat tissue (6). In our *in vivo* study on the effect of HI treatment for 4 weeks on thyroid tissue revealed a significant increase in pERK1/2, but no effect on pAkt level. Thus, Akt phosphorylation may not be the primary signaling pathway that mediates thyroid cell proliferation after HI administration, and pERK1/2 may play a greater role, however further studies are needed to clarify this hypothesis.

HI and glargine treatment produced a higher phosphorylation of ERK1/2 than control control, with glargine having slightly stronger effects compared to HI. According to insulin cell-signaling pathways, this could be due to the higher affinity between glargine and IGF-1R relative to IR (20), because ERK1/2 is the primary downstream effector of IGF-1R. Previous studies also confirmed (37, 38) that ERK1/2 and MEK/ERK1/2 pathways are involved in cell survival, proliferation, differentiation, and mitosis. Kandil et al. (39) found that inhibition of MEK/ERK expression exerts cytotoxic effects on thyroid cancer cells. Our study on pERK1/2 activation after insulin administration may be pertinent to more than the role of hypoglycemic drugs, but could also shed light on mechanisms of increased thyroid cell proliferation. The further experiments are needed to characterize the effects of insulin and its analogs on thyroid cell proliferation *in vivo* and *in vitro*. It also suggests that clinicians should consider the possibility that insulin may stimulate thyroid cell proliferation during the treatment of diabetes.

## Conclusion

Our *in vivo* experiments show that therapeutic doses of glargine have a longer-lasting hypoglycemic effect than HI, and promote the phosphorylation of IR, Akt, and ERK1/2. High doses of HI and glargine can promote thyroid cell proliferation, while therapeutic doses of HI and glargine do not cause thyroid cell proliferation. HI may be associated with IR phosphorylation, while glargine may be associated with IGF-1 phosphorylation. Both receptors can activate PI3K-Akt and MAPK signaling pathways to promote proliferation.

## Data Availability

All datasets generated for this study are included in the manuscript and/or the supplementary files.

## Ethics Statement

This study was carried out in accordance with the recommendations of Zhejiang Chinese Medical University of guidelines of committee. The protocol was approved by the Zhejiang Chinese Medical University of committee.

## Author Contributions

XS, KY, AS, ST, and DY substantially contributed to the conception and design of this study, drafted the work, gave final approval of the version to be published, and agreed to be accountable for all aspects of this work and ensured that issues pertaining to the accuracy or integrity of any part of this work are appropriately investigated and resolved. XZ and TC critically revised the manuscript for important intellectual content.

### Conflict of Interest Statement

The authors declare that the research was conducted in the absence of any commercial or financial relationships that could be construed as a potential conflict of interest.
